# Serological and Molecular Identification of *Brucella* spp. in Pigs from Cairo and Giza Governorates, Egypt

**DOI:** 10.3390/pathogens8040248

**Published:** 2019-11-20

**Authors:** Aman Ullah Khan, Falk Melzer, Sherif Abdel Ghafar Elsayed El-Soally, Mandy C. Elschner, Shereen Aziz Mohamed, Mohamed Abdelmonem Sayed Ahmed, Uwe Roesler, Heinrich Neubauer, Hosny El-Adawy

**Affiliations:** 1 Friedrich-Loeffler-Institut, Institute of Bacterial Infections and Zoonoses, 07743 Jena, Germany; AmanUllah.Khan@fli.de (A.U.K.); falk.melzer@fli.de (F.M.); mandy.elschner@fli.de (M.C.E.); Heinrich.neubauer@fli.de (H.N.); 2 Institute for Animal Hygiene and Environmental Health, Free University of Berlin, 14163 Berlin, Germany; Uwe.Roesler@fu-berlin.de; 3 Department of Pathobiology, College of Veterinary and Animal Sciences (Sub-Campus UVAS-Lahore), Jhang 35200, Pakistan; 4 Veterinary Service Department, Armed Forces Logistics Authority, Egyptian Armed Forces, 11765 Nasr City, Egyptmoh_vet2001@hotmail.com (M.A.S.A.); 5 Veterinary Serum and Vaccine Research Institute, 11517 Abbasaia, Cairo, Egypt; shereenibraheem1968@gmail.com; 6 Faculty of Veterinary Medicine, Kafrelsheikh University, 33516 Kafr El-Sheikh, Egypt

**Keywords:** brucellosis, swine, Egypt, ELISA, real-time PCR

## Abstract

Brucellosis is considered as endemic disease of animals and humans since thousands of years in Egypt. However, brucellosis in pigs has never been reported in Egypt. Thus, serological and molecular assays were applied to detect anti-*Brucella* antibodies and DNA in serum samples collected from pigs. In total 331 blood samples collected from male and female pigs at slaughterhouses of Cairo and Giza governorates were investigated using *Brucella* c- and i-ELISA and *Brucella* real-time PCR. Anti-*Brucella* antibodies were detected in 16 (4.83%) and 36 (10.8%) sera by i-ELISA and c-ELISA, respectively. *Brucella* DNA was detected in 10 (3.02%) seropositive samples and identified as *Brucella melitensis* (7/10) *and Brucella suis* (3/10). A higher prevelance was found in boars. This is the first study investigating pig brucellosis in Egypt. The results of this study will raise awareness for brucellosis in these farm animals and will help to develop effective control strategies.

## 1. Introduction

Brucellosis is a zoonotic disease of public health importance affecting livestock, wildlife, and humans globally. The *Brucella* (*B.*) genus includes eleven recognized species with varying host preferences, pathogenicity, and epidemiology [1,2]. The disease is well controlled in developed countries but is still endemic in many others with the highest records in humans in Middle East and Central Asian regions [3]. 

Brucellosis is one of the major livestock production constraints in Egypt [4]. It is likely that it has been endemic in Egypt for thousands of years [5,6]. The disease has been detected with increasing prevalence in livestock species but predominantly in ruminants [7,8]. Prevalences ranging from 2.47% to 26.66% were found in various animal populations [9]. *Brucella abortus* and *B. melitensis* were isolated from livestock and humans and *B. suis* was identified in cattle [6,10]. Brucellosis proved to be a serious occupational health hazard to livestock handlers, especially abattoir workers in Egypt [11]. 

World pig production has increased fourfold over the last five decades to meet protein requirements globally and is expected to continue growing [12]. Production of pigs in Egypt is found primarily in slums, rural, and per-urban areas especially in Cairo and Giza governorates. Pork is consumed by Christians, foreigners, and tourists in Egypt. Currently the pig population is around two to three millions [13,14]. In Egypt, pigs are kept in small groups in contact with other farm animals and humans sharing pathogens with each other [15]. 

Typically, the infection is caused by *B. suis* biovars 1–3 [16]. The disease occurs in many countries where pigs are raised. Generally, the prevalence is low, but in some parts of the world, especially in the Southeast Asia and the South America, the prevalence may be much higher. *B. suis* bv1 infection has been reported in feral pigs in some parts of the southern states of USA and in Queensland, Australia. In these regions, a number of human brucellosis cases have been reported in hunters and handlers of materials of feral pigs. *B. suis* bv2 outbreaks have also been reported in Europe in wild boars, which were implicated in transmission of *B. suis* bv2 to domestic outdoor pigs [2]. Human pathogenic biovars (*B. suis* biovar 1–4) pose a sever hazard to humans [16]. Hence, *B. abortus* [17,18] and *B. melitensis* [19,20] were also isolated from pigs when kept together with infected ruminants and camels. 

Brucellosis in pigs is a contagious disease characterized by infertility, production of small litters, and abortion in sows and orchitis and infection of secondary sex organs in boars [21]. The clinical manifestations are not pathognomonic. A diagnosis of brucellosis can be made mainly by the isolation and identification of *Brucella*, but in situations where bacteriological examination is not practicable, diagnosis should be based on immunological methods [2]. Serological tests are preferred for screening as they are comparatively sensitive and specific compared to bacterial cultivation to minimize the risk of laboratory acquired infections [2]. 

“Pig” brucellosis in humans is frequently a disease of slaughterhouse workers, farmers, and veterinarians [16]. Direct contact with infected animals or aborted materials may lead to human infection. In humans, brucellosis is generally a chronic illness manifested by intermittent fever, malaise, night sweats, and musculoskeletal and neurological signs [2,16]. 

For serological testing various tests, usually a screening test of high sensitivity, followed by a confirmatory test of high specificity are used [22]. Generally, c-ELISAs are more specific than i-ELISAs but less sensitive [23]. Sensitivities and specificities of ELISAs were evaluated previously showed that 100% sensitivity and specificity were found for c-ELISAs, and i-ELISA showed 99.1% specificity and 100% sensitivity, respectively [24]. c-ELISAs proved to be highly sensitive and specific when compared to other commonly used serological tests, i.e., Rose Bengal test, fluorescence polarization assay, i-ELISA for diagnosis of swine brucellosis [25]. 

Although confirmation of the disease is achieved by bacterial culture and isolation of the etiological agent, *Brucella* is difficult to grow and bacterial culturing and biochemical identification are time consuming. Additionally, this method poses risk to laboratory personnel and requires specific biosafety measures [26]. Hence, detection of DNA by PCR in clinical samples is considered a preferred tool for definitive diagnosis of brucellosis [27]. 

Although brucellosis in pigs has not been noted in Egyptian surveillance reports, a Rose Bengal plate agglutination assay (RBPT) was performed previously to quantify the risk for workers in slaughterhouses [15]. 

Considering public health concerns and the zoonotic importance of brucellosis, the present study was aimed to identify seropositive pigs at slaughterhouses and to characterize subsequently the *Brucella* species involved in swine brucellosis in Egypt. 

## 2. Materials and Methods

### 2.1. Study Area and Sera Collection

The study was conducted from March 2017 to July 2019. The serum samples were collected from abattoirs of Cairo and Giza governorates in Egypt. The data for each sample including origin, sex, and date of sampling were recorded. In total, 331 blood samples (116 from males and 215 from females) were collected in sterile vacutainer tubes without anticoagulant. The serum was harvested and stored at −20 °C. The serum was shipped to Friedrich–Loeffler Institut, Jena, Germany for further analysis.

### 2.2. Ethics Statement

This study was carried out in strict accordance with the recommendations in the Guide of the Egyptian Network of Research Ethics Committees (ENREC), which complies with the international laws and regulation regarding ethical considerations in research. All efforts were made to minimize animal suffering and to reduce the number of animals used.

### 2.3. Detection of Anti-Brucella Antibodies

Antibody detection was carried out using the IDVet indirect ELISA kit (ID Screen^®^ Brucellosis Serum Indirect Multi-species) (IDVet Innovative Diagnostics Grabels, France) and the SVANOVIR^®^ *Brucella*-Ab c-ELISA kit (Uppsala, Sweden) according to the manufacturer’s instructions. 

### 2.4. Molecular Detection of Brucella DNA

DNA was extracted from all collected serum samples by using the QIAamp DNA Mini Kit (QIAGEN, Germany) according to the instructions of the manufacturer. 

Genus- (*Brucella*) and species-specific (*B. abortus, B. melitensis*, and *B. suis*) multiplex real-time PCRs were used for detection of *Brucella* DNA. PCR was performed using the primer and probe sets given in Table 1 (Jena Bioscience GmbH, Germany). Briefly, the PCR reaction was done in a 15 μL multiplex PCR mixture with 2× TaqMan™ Environmental master mix (Applied Biosystems^®^, Germany), 0.2 µM of each primer, 0.1 µM of each probe, and 5 μL of template DNA. Amplification and real-time fluorescence detection was carried out on a StepOnePlus™ Real-Time PCR System (Applied Biosystems^®^, Germany). The reaction conditions were decontamination at 50 °C for 2 min, initial denaturation at 95 °C for 10 min followed by 50 cycles of denaturing at 95 °C for 25 s and annealing/elongation at 60 °C for 1 min. Sample data scores were confirmed by visual inspection of graphical plots and cycle threshold (CT) values for each sample were obtained. CT values below 38 were considered positive. Reference strains of *B. abortus* S-99 (ATCC 23448), *B. melitensis* 16M (ATCC 23456), and *B. suis* biovar 1 (ATCC 23444) were used as positive controls for each PCR reaction to ensure no cross-contamination. 

## 3. Results 

### 3.1. Anti-Brucella Antibodies in Pig Sera

Out of 331 sera samples, 16 (4.83%) were positive for anti-*Brucella* antibodies by i-ELISA and 36 (10.8%) were positive by c-ELISA. In the Cairo governorate, 1.21% and 9.75% sera were positive while, in the Giza governorate, 6.02% and 11.2% were positive by i-ELISA and c-ELISA, respectively. Anti-*Brucella* antibodies were detected in 12.9% and 21.5% of boars by i-ELISA and c-ELISA, respectively. Only 1 (0.46%) female animal was seropositive by i-ELISA while 5.11% were positive by c-ELISA (Table 2). Only three sera samples were positive with both ELISAs. 

### 3.2. Detection of Brucella DNA in Pig Sera

*Brucella*-specific DNA was detected in 10 (3.02%) samples and typed as *B. melitensis* (7/10) and *B. suis* (3/10) (Table 2). In Cairo, 3.65% sera were positive, and in Giza it was 2.81%. In 6.03% boars *Brucella*-specific DNA was detected, in female pigs it was 1.39%. Boars originating from Giza governorate were more often positive (11.7%) than those from the Cairo governorate (3.65%). Only three sera were positive with all tested assays, while *Brucella* DNA was detected in all c-ELISA positive serum samples. 

## 4. Discussion

This study is the first investigation of swine brucellosis using serological and molecular tools in Egypt. Despite the endemicity of *Brucella* infection in humans and ruminants for many years [7], pig brucellosis has never been reported. Many published studies highlighted the identification of *Brucella* in various animal species (cattle, buffalo, sheep, goat, bison, African buffalo and Alpine ibex) to define their potential role in disease spread [30,31,32,33]. The pigs investigated in this study were raised in slums, rural, and per-urban areas likely having close contact with other livestock (cattle, sheep, and goats) which may lead to sharing of pathogens with each other as described previously in Egypt [15]. 

Swine brucellosis is a zoonotic disease and is widely prevalent in many pig-rearing countries [16]. The proof of the existence of swine brucellosis in Egypt may now raise awareness and can help to tailor control strategies to improve human health. 

Brucellosis is diagnosed usually by using serological screening tests of high sensitivity followed by highly specific tests due to the false–positive reactions which probably arise from cross-reactions with other bacteria and mainly with *Yersinia enterocolitica* O:9. Swine serum may sometimes contain nonspecific antibodies, probably IgM, that reduce the specificity of conventional tests, especially for serum agglutination tests. Moreover, the swine complement interacts with the guinea-pig complement to produce pro-complementary activity that reduces the sensitivity of the complement fixation test (CFT) [2]. The c-ELISA is more sensitive and specific in swine brucellosis serology [25]. Both serological tests applied showed different results. Previous studies on pig brucellosis found 100% sensitivity and specificity for c-ELISA, and 99.1% specificity and 100% sensitivity for the i-ELISA, respectively [24]. 

In this study, 331 samples of pigs collected at slaughterhouses of Cairo and Giza governorates that have the highest swine populations in Egypt were investigated. A higher number of seropositive pigs was recorded by c-ELISA (10.8%) when compared to the i-ELISA (4.83%). Although these samples were not taken following the sampling plan of the Egyptian prevalence study plan of ruminants, the ranges are in agreement with the previous prevalence reports of brucellosis in cattle, buffaloes, sheep, and goats in Egypt [7,10,34]. 

Quantitative real-time PCR for *Brucella* DNA detection has proved highly specific and sensitive when compared to other conventional PCR assays and serology [35]. In the current study, *Brucella* DNA was detected in 3.02% of pig samples. Qualitative multiplex real-time PCR confirmed seven *B. melitensis* and three *B. suis* DNAs. Detection of *B. melitensis* DNA in the present study in pigs reared in Cairo and Giza governorates was expected as previous reports showed the endemicity of *B. melitensis* in these regions in Egypt [7]. The identification of *B. suis* in this study is not unexpected as *B. suis* was previously isolated from cattle [6] ensuring the presence of these species in Egypt. The detection of a higher number of *B. melitensis* DNA samples as compared to *B. suis* DNA in this study is expected as these pigs are in close contact with free grazing sheep and goat flocks. It is common in extensive livestock farming to share pastures and watering. Such type of mixing of animals is an important risk factor to spread the disease from infected to healthy animals or other livestock species [36]. Most sheep and goat flocks are mobile in Egypt. Movements of infected animals can contaminate feeding and grazing areas and may spread infection to other animals (e.g., cattle, buffalo or camel) [10]. The prevalence of *B. melitensis* and *B. suis* in swine may be attributed to the cross-contamination or co-rearing of pigs with other animals [6,15,19,20].

In this study, *Brucella* DNA was detected in 10 (3.02%) of the seropositive samples. Out of 10 positive DNAs, three samples were found positive with both ELISAs (i-ELISA and c-ELISA), while seven DNAs were found positive in samples which were only positive with c-ELISA. The higher number of *Brucella* DNA was identified in c-ELISA positives sera. It was proven that c-ELISA has shown higher sensitivities and specificities for the diagnosis of swine brucellosis [25].

Sex dependent prevalence has been documented in cattle and small ruminants, i.e., *B. melitensis* is more often found in females. Hence, this phenomenon in pigs has not fully been investigated, it may affect both sexes (male and female) equally [37]. In this study, higher prevalence was found in male pigs than in female pigs. Significantly higher molecular prevalence of brucellosis in males (27.7%) than in females (8.09%) were previously reported from India [21]. Higher prevalence of anti-*Brucella* antibodies in boars was reported also 11.11% vs 3.29% from Nepal, previously [38]. 

The endemic nature of the disease, particularly the identification of *B. melitensis* and *B. suis* DNA from swine sera suggests a complex underlying epidemilogical situation in Egypt. 

## 5. Conclusions

To the best of our knowledge, this is the first study reporting the presence of anti-*Brucella* antibodies and *Brucella* DNA in serum collected from pigs in Egypt. This study, although performed on a limited number of samples and focusing on two governorates only, gives an insight on the situation of brucellosis and *Brucella* species prevalent in pigs in Egypt. As the investigated pigs in this study were apparently healthy and admitted for slaughtering, we believe that pigs can be carriers of brucellosis and present a risk to livestock or even humans or may act as a dead-end host, unlikely to be involved in the transmission. Further investigation is needed to assess the prevalence of *Brucella* species particularly *B. suis* in swine to explore the ways of cross-contamination and the risk for consumers. 

## Figures and Tables

**Table 1:** Primer and probe sequences used in real-time multiplex PCR assay for the detection of *Brucella* spp., *B. abortus, B. melitensis*, and *B. suis*.

Target	Primer sequences		Reference
*Brucella*	5’-GCT CGG TTG CCA ATA TCA ATG C-3′ 5′-GGG TAA AGC GTC GCC AGA AG-3′ FAM-AAA TCT TCC ACC TTG CCC TTG CCA TCA-MGB	Forward Reverse Probe	[28]
*B. abortus*	5′-GCG GCT TTT CTA CGG TAT TC-3′ 5′-CAT GCG CTA TGA TCT GGT TAC G-3′ Joe-CGC TCA TGC TCG CCA GAC TTC AAT G-BHQ-1	Forward Reverse Probe	
*B. melitensis*	5′-AAC AAG CGG CAC CCC TAA AA-3′ 5′-CAT GCG CTA TGA TCT GGT TAC G-3′ NED-CAG GAG TGT TTC GGC TCA GAA TAA TCC ACA-MGB	Forward Reverse Probe	
*B. suis*	5′-GCC AAA TAT CCA TGC GGG AAG-3′ 5′-TGG GCA TTC TCT ACG GTG TG-3′ VIC-TTGCGCTTTTGTGATCTTTGCTTATGG-MGB	Forward Reverse Probe	[29]

**Table 2:** Seroprevalence and molecular identification of *Brucella*-DNA in pig sera collected from Cairo and Giza governorates, Egypt.

Governorates	Sex	Number of Samples	Number of Brucellosis Positive Sera		Molecular Identification		
			i-ELISA No. (%)	c-ELISA No. (%)	Real-Time PCRNo. (%)	*Brucella* spp. DNA	*Ct* value
Cairo	male	82	1 (1.21)	8 (9.75)	3 (3.65)	*B. melitensis*	34
						*B. melitensis*	34
						*B. melitensis*	36
Giza	male	34	14 (41.1)	17 (50.0)	4 (11.7)	*B. melitensis*	36
						*B. melitensis*	29
						*B. suis*	36
						*B. suis*	34
	female	215	1 (0.46)	11 (5.11)	3 (1.39)	*B. melitensis*	36
						*B. melitensis*	36
						*B. suis*	36
Total		331	16 (4.83)	36 (10.8)	10 (3.02)		
